# Human Adult Renal Progenitor Cells Prevent Cisplatin-Nephrotoxicity by Inducing CYP1B1 Overexpression and miR-27b-3p Down-Regulation through Extracellular Vesicles

**DOI:** 10.3390/cells12121655

**Published:** 2023-06-17

**Authors:** Rossana Franzin, Alessandra Stasi, Giuseppe De Palma, Angela Picerno, Claudia Curci, Serena Sebastiano, Monica Campioni, Antonella Cicirelli, Alessandro Rizzo, Vito Francesco Di Lorenzo, Paola Pontrelli, Giovanni Battista Pertosa, Giuseppe Castellano, Loreto Gesualdo, Fabio Sallustio

**Affiliations:** 1 Renal, Dialysis and Transplantation Unit, Department of Precision and Regenerative Medicine and Ionian Area (DiMePRe-J), University of Bari, 70124 Bari, Italy; alessandra.stasi@uniba.it (A.S.); claudiacurci@gmail.com (C.C.); s.sebastiano1@studenti.uniba.it (S.S.); monica.campioni@uniba.it (M.C.); paola.pontrelli@uniba.it (P.P.); giovannibattista.pertosa@uniba.it (G.B.P.); loreto.gesualdo@uniba.it (L.G.); 2 MIRROR—Medical Institute for Regeneration, Repairing and Organ Replacement, Interdepartmental Center, University of Bari Aldo Moro, 70124 Bari, Italy; 3 Institutional BioBank, Experimental Oncology and Biobank Management Unit, IRCCS Istituto Tumori “Giovanni Paolo II”, 70124 Bari, Italy; g.depalma@oncologico.bari.it; 4 Department Interdisciplinary of Medicine (DIM), University of Bari, 70124 Bari, Italy; angela.picerno@uniba.it (A.P.); antonella.cicirelli@uniba.it (A.C.); 5 Struttura Semplice Dipartimentale di Oncologia Medica per la Presa in Carico Globale del Paziente Oncologico ‘Don Tonino Bello’, IRCCS Istituto Tumori ‘Giovanni Paolo II’, Viale Orazio Flacco 65, 70124 Bari, Italy; rizzo.alessandro179@gmail.com; 6 Urology Unit, IRCCS Istituto Tumori “Giovanni Paolo II”, 70124 Bari, Italy; vitodilorenzo1957@gmail.com; 7 Unit of Nephrology, Dialysis and Renal Transplantation, Fondazione IRCCS Ca’Granda Ospedale Maggiore Policlinico di Milano, 20122 Milan, Italy; giuseppe.castellano@unimi.it

**Keywords:** cisplatin-induced AKI, onconephrology, regenerative medicine, tubular adult renal progenitor cells, extracellular vesicles, CYP1B1, cytochrome P450, miR-27b-3p, MIR210HG, LINC00511

## Abstract

Cisplatin is one of the most effective chemotherapeutic agents strongly associated with nephrotoxicity. Tubular adult renal progenitor cells (tARPC) can regenerate functional tubules and participate in the repair processes after cisplatin exposition. This study investigated the molecular mechanisms underlying the protective effect of tARPC on renal epithelium during cisplatin nephrotoxicity. By performing a whole-genome transcriptomic analysis, we found that tARPC, in presence of cisplatin, can strongly influence the gene expression of renal proximal tubular cell [RPTEC] by inducing overexpression of CYP1B1, a member of the cytochrome P450 superfamily capable of metabolizing cisplatin and of hypoxia/cancer-related lncRNAs as MIR210HG and LINC00511. Particularly, tARPC exerted renoprotection and regeneration effects via extracellular vesicles (EV) enriched with CYP1B1 and miR-27b-3p, a well-known CYP1B1 regulatory miRNA. The expression of CYP1B1 by tARPC was confirmed by analyzing biopsies of cisplatin-treated renal carcinoma patients that showed the colocalization of CYP1B1 with the tARPC marker CD133. CYP1B1 was also overexpressed in urinary EV purified from oncologic patients that presented nephrotoxicity episodes after cisplatin treatment. Interestingly CYP1B1 expression significantly correlated with creatinine and eGFR levels. Taken together, our results show that tARPC are able to counteract cisplatin-induced nephrotoxicity via CYP1B1 release through EV. These findings provide a promising therapeutic strategy for nephrotoxicity risk assessment that could be related to abundance of renal progenitors.

## 1. Introduction

Cisplatin is an inorganic platinum-based chemotherapeutic agent widely used in the treatment of a variety of solid malignant tumors, such as head and neck, lung, testis, ovarian, and bladder cancers. Even though it has potent anticancer properties and efficacy, cisplatin has severe adverse side effects, including ototoxicity, neurotoxicity and nephrotoxicity [1,2,3].

Cisplatin nephrotoxicity can manifest in several ways, but the most severe and common manifestation is acute kidney injury (AKI), which occurs in 20–30% of patients [4]. Typically, AKI results in acute tubular necrosis, which may progressively deteriorate kidney function and can lead to chronic kidney disease (CKD) [5]. The pathophysiology of cisplatin-induced nephrotoxicity and AKI involves a multitude of mechanisms, such as necrosis and apoptosis, oxidative stress, autophagy, dysregulation of cell cycle proteins, DNA and mitochondrial DNA damage, endoplasmic reticulum stress, and inflammation [1,2,6]. Cisplatin is transported into kidney cells via organic cation transporters 2 and copper transporter 1 and undergoes biotransformation in highly reactive thiols in proximal tubular cells through the formation of cysteine conjugate [4,5,7,8,9]. Following a cisplatin treatment, a five-fold increase in cisplatin concentration was observed in kidney tissue compared to other tissue, indicating accumulation in renal cells [10]. Today, alleviation of cisplatin induced-nephrotoxicity is accomplished by short-term and lower-volume hydration, magnesium supplement, or mannitol-induced forced diuresis. However, mannitol treatment causes over-diuresis and consequent dehydration in cisplatin-treated patients, highlighting the urgent need for the clinical implementation of new, effective renoprotective strategies [5]. During episodes of AKI, the capability of the kidney to regenerate functional tubules is supported by surviving renal proximal tubular epithelial cells (RPTEC), which proliferate and migrate to replace the neighboring injured cells [11,12,13,14]. Human tubular adult renal progenitor/stem cells (tARPC), a population of tubule progenitor cells present in the renal tubules, can also replace necrotic and apoptotic cells in response to injury by proliferating and differentiating into the various renal cell types [11,12,15,16,17]. Interestingly, tARPC are cisplatin-resistant and can regenerate cisplatin-induced chemical damage through the secretion of protective molecules and extracellular vesicles [12,16,18,19]. In order to provide insight into molecular mechanisms associated with tARPC-mediated resistance to cisplatin we performed a whole genome transcriptomic analysis of RPTEC after cisplatin exposition with or without a co-culture with tARPC. 

Here we show that tARPC act as sentinels of cisplatin-damaged RPTEC and exert a renoprotective role by inducing overexpression of the CYP1B1 gene, a cytochrome p450 member implicated in the metabolism of a diverse range of drugs including tamoxifen, docetaxel, and—importantly—cisplatin. Furthermore, we demonstrated that this process is mediated by CYP1B1-loaded extracellular vesicles, possibly released by tARPC in response to cisplatin RPTEC damage, also in oncological patients with cisplatin-induced nephrotoxicity. 

## 2. Materials and Methods

### 2.1. Co-Culture Experiments

Human CD133^+^ tARPC were purified from healthy parts of kidney cortexes from patients undergoing nephrectomy for renal clear-cell carcinoma, cellular immunophenotyping was assessed as previously described [12]. The study was carried out according to the principles of the Declaration of Helsinki and was approved by the IRCCS Istituto Tumori “Giovanni Paolo II”. All patients signed an informed written consent form for leftover material for scientific purposes approved by the local ethics committee of the IRCCS Istituto Tumori “Giovanni Paolo II” of Bari (protocol no. 8/CE of 2 February 2021). All patients presented written informed consent for the use of this material for research purposes. After isolation, tARPC were grown in endothelial cell growth medium (EGM) (Lonza, Basel, Switzerland) supplemented with 20% fetal bovine serum (FBS) at 37 °C and 5% CO_2_. Human RPTEC were purchased from ATCC-LGC (ATCC-LGC Standards S.r.l., Sesto San Giovanni, Milan, Italy) and Lonza (Lonza, Basel, Switzerland). RPTEC were maintained in the recommended medium REGM (Lonza) with 100 U/mL penicillin and 100 ng/mL streptomycin. The RPTEC medium was serum-free, and all RPTEC and tARPC co-cultures were performed in RPTEC medium. For in vitro experiments, RPTEC were plated at a density of 10,000 cells/cm^2^, and 48 h later, they were incubated in medium alone or in the presence of 2.5 mmol/L cisplatin for 6 h. For co-culture experiments, we used our in vitro cisplatin-induced cell injury model as previously reported [12]. Briefly, tARPC were seeded on top of 0.4 mm thick polycarbonate inserts (Transwells) (Costar Corning, Life Sciences, Acton, MA, USA) at 8000 cells/cm^2^ in RPTEC medium and were used for co-cultures after 2 days of serum-free medium condition. RPTEC were damaged with 2.5 mmol/L cisplatin for 6 h and after 24 h they were co-cultured with tARPCs on the Transwells for four days. We then analyzed the following conditions: (1) RPTEC basal conditions (RPTEC Bas), RPTEC grown in basal conditions for 96 h; (2) RPTEC damaged by cisplatin for 6 h (RPTEC Cis) followed by medium refresh and growth for 96 h; (3) RPTEC damaged by cisplatin for 6 h followed by co-culture with tARPC for 96 h (RPTEC co-culture); (4) tARPCs basal conditions for 96 h; (5) tARPCs damaged by cisplatin for 6 h (tARPCs Cis) followed by medium refresh and growth for 96 h; (6) tARPCs from co-culture (from Condition 3).

### 2.2. Evaluation of Apoptosis

For apoptosis analysis, Annexin-V and 7AAD staining (Beckman coulter, Milan, Italy) was performed according to the manufacturer’s instructions. Data were obtained using an FC500 flow cytometer (Beckmann Coulter) and analyzed using Kaluza software.

### 2.3. Caspase-3 Staining

The cells were blocked for 1 h (BSA in PBS, pH 7.4) and then incubated with primary antibodies overnight at 4 °C or for 2 h at room temperature. The immune complexes were identified with the respective specific secondary antibodies for 1 h at room temperature. For caspase-3, cells were permeabilized with Triton 0.25% for 5 min, then blocked with protein block solution (Dako Cytomation, Santa Clara, CA, USA) for 10 min. Incubation was performed with antibodies against: caspase-3 (Novus Biologicals, Abingdon Science Park, Oxford, UK) and detected using the Peroxidase/DAB Dako Real EnVision Detection System (Dako, Glostrup, Denmark). The peroxidase reaction was shown by a brown precipitate counterstained with Mayers hematoxylin (blue). Negative controls were prepared via incubation with a control-irrelevant antibody. Images were scanned using an Aperio ScanScope CS2 device, and signals were analyzed with the ImageScope V12.1.0.5029 (Aperio Technologies, Vista, CA, USA).

### 2.4. Transcriptomic Profile

Total RNA was extracted from the cultures using RNeasy Mini Kit (Qiagen, Germantown, MD, USA) according to the protocol described by the manufacturer and stored at −80 °C. The concentrations of extracted RNA were checked using a UV–Vis spectrophotometer (NanoDrop 2000), and the quality of the extracted data was analyzed using an RNA 6000 Nano Assay Kit (Agilent, Santa Clara, CA, USA) and a BioAnalyzer 2100 (Agilent, USA). RNA samples with RIN (RNA integrity number) values higher than 8 were used in transcriptomic analysis. DNA microarray analysis was performed on the Agilent SurePrint G3 Human gene expression v3 microarray, including long non-coding RNA (lncRNA) probes covering the LNCipedia 2.1 database and updated mRNA probes. 

A one-color RNA spike-in kit (Agilent, ABD) was used to provide internal controls with known concentrations. The total RNA samples were then mixed with diluted RNA spike-in controls and labelled with cyanine 3 (Cy3) using a low-input quick amp labeling Kit (Agilent, USA). The Cy3-labelled samples were purified using an Absolutely RNA Nanoprep Kit (Agilent, USA) and incubated on microarray slides (RNA 6000 NanoLabChip^®^ Kit, Agilent, USA). The microarray slides were transferred into a microarray hybridization chamber (Agilent, USA) for hybridization at 65 °C for 17 h. Subsequently, the slides were washed and transferred into an Agilent C scanner device according to the manufacturer’s instructions. Transcriptomic data analysis was performed using GeneSpring Data Analysis Software. Reliability of the gene expression data was assessed via application of Student’s *t*-test and Benjamini and Hochberg (1995) or Storey *p* value correction using corrected *p* values (false discovery rate, FDR) lower than 0.05 and a fold change (FC) >2. 

The Agilent microarray data are Minimum Information About a Microarray Experiment (MIAME)-compliant, and raw data have been deposited in the database of the European Bioinformatics Institute (EMBL-EBI) and are accessible through Experiment ArrayExpress accession number E-MTAB-11451.

### 2.5. RNA Extraction and RT-PCR

Co-cultured cells were lysed for RNA extraction using an RNeasy Mini Kit (Quiagen) following manufacturer’s instructions. The RNA concentration was determined with a NanoDrop Spectrophotometer (Nanodrop Technologies, Wilmington, DE, USA). RT-PCR was performed using an iScript cDNA Synthesis Kit (Bio-Rad) according to the manufacturer’s instructions. 

### 2.6. Real-Time PCR

Real-time PCR mix was prepared by SsoAdvanced™ Universal SYBR^®^ Green Supermix (Biorad) for the target BAX and CYP1B1, gene expression was assessed using Light Cycler@96 (Roche). The relative amounts of mRNA were normalized to β-actin mRNA as the housekeeping gene. Data were analyzed using the ΔΔCt method. Primer sequences and other details used are given in Table S1 (Supplementary Materials).

### 2.7. lncRNA qPCR

RT^2^ lncRNA qPCR assays were designed for SYBR^®^-Green-based real-time PCR detection of long, non-coding RNAs from Qiagen. Specifically, MIR210HG was analyzed using assay LPH15919A, and the target, LINC00511, was evaluated with assay LPH06350A. 

Briefly, 5 ug of total RNA were used after genomic DNA elimination for RT2 First Strand cDNA reverse transcription, then RT^2^ SYBR Green Mastermix, RT^2^ IncRNA qPCR Assay, and cDNA synthesis reaction were used for qPCR. 

### 2.8. miRNA Analysis

The TaqMan-based real-time PCR method was used to evaluate the expression of mir27b-3p. The protocol performed was the TaqMan^®^ advanced miRNA single-tube assay (catalog number A25576, pub. No. 100027898, Rev. single-tube assays). Briefly, 2 μL of RNA from EV sample was reverse-transcribed with the Applied Biosystems™ TaqMan™ Advanced miRNA cDNA Synthesis Kit (ThermoFisher Scientific, Dreieich, Germany). TaqMan RT-PCR was performed with Applied Biosystems™ TaqMan™ Advanced miRNA Assays (ThermoFisher Scientific, Germany) and TaqMan1Universal MasterMixII (ThermoFisher Scientific, Germany) for candidate targets and endogenous control, respectively. hsa-miR-16-5p was used for normalization (ThermoFisher Scientific, Germany) according to the formula 2-DDCT. RNA extraction was performed from culture supernatant EV using an miRNeasy mini kit (Qi-agen). The EV total RNA quantification was assessed using NanoDrop 1000 and the Qubit 2.0. Fluorometer as already demonstrated [20]. DNase treatment was carried out to remove any contaminating DNA (RNase-Free DNase Set, Qiagen). The RNA concentration was determined with a NanoDrop Spectrophotometer (Nanodrop Technologies, Wilmington, DE, USA). RNA contained in EVs was purified, analyzed in quality using Bioanalyzer (RNA nano), and evaluated with qPCR. Data were analyzed using the ΔΔCt method. Primer sequences and other details used are given in Table S1 (Supplementary Materials).

### 2.9. miRNA Target Analysis

Regulatory miRNA of CYP1B1 genes were detected by applying several computational algorithms. To confirm validated and predicted interactions between CYP1B1 mRNA and miR-27b-3p we used: TargetScan 5.2 (http://www.targetscan.org, accessed on 10 March 2023), miRTarBase47 and DIANA TarBase v7.04, and miRBase 17.0 (http://microrna.sanger.ac.uk, accessed on 10 March 2023).

### 2.10. EV Extraction from tARPC/RPTEC Co-Culture Supernatants

The EV isolation from co-culture supernatant was performed by means of a miRCURY Exosome Cell/Urine/CSF Kit (Qiagen) following the manufacturer’s instructions.

### 2.11. Urinary EV Purification from Oncological Patients

The study was conducted according to the guidelines of the Declaration of Helsinki and approved by the Ethics Committee of Istituto Tumori “Giovanni Paolo II”, Bari (protocol code 8, date of approval 2 February 2021). Informed consent was obtained from all subjects involved in the study. Clinical data of patients enrolled in the study are indicated in Table 1. Total RNA from EV was isolated from 30 mL of urine samples of cisplatin-treated patients, and the exoRNeasy Midi Kit (QIAGEN, Hilden, Germany) was used according to the manufacturer’s instructions. The extracellular RNA was eluted in 14 µL of RNase-free water and quantified using a NanoDrop One Spectrophotometer (Thermo Fisher, Waltham, MA, USA).

### 2.12. KIM-1 ELISA

Urinary kidney injury molecule-1 (KIM-1) analysis was performed using commercially available ELISA kits (MyBioSource, San Diego, CA, USA) according to the manufacturer’s instructions.

### 2.13. Immunofluorescence Staining

#### 2.13.1. tARPCs Immunofluorescence

After being cultured and after 6 h incubation with cisplatin at 2.5 mmol/L, tARPCs were fixed in formalin for 15 min and blocked for 30 min (BSA in PBS, pH 7.4). Next, cells were incubated with primary antibodies overnight at 4 °C. The immune complexes were identified with the respective specific secondary antibodies for 1 h at room temperature. The following antibodies were used: rabbit polyclonal to CYP1B1 (ab137562, Abcam). The following secondary antibodies were used: Alexa Fluor 555 goat anti-rabbit IgG.

#### 2.13.2. RCC Immunofluorescence

Paraffin-embedded human RCC sections were deparaffinized, rehydrated, treated for antigen retrieval, and incubated in blocking solution prior to incubation with primary antibodies at room temperature (RT) at 4 °C overnight. The immune complexes were identified with the respective specific secondary antibodies for 2 h at room temperature. The following primary antibodies were used: rabbit polyclonal CYP1B1 (ab137562, Abcam), mouse anti-human monoclonal CD133/2 (pure, 120-001-247, Miltenyi Biotec). The following secondary antibodies were used: Alexa Fluor 555 goat anti-rabbit IgG, Alexa Fluor 488 goat anti-mouse IgG. Human CD133 (Prominin-1) mouse anti-human monoclonal Ab (TMP4) PE (#12-1338-42,Invitrogen) was used to perform direct immunolabeling. All cells and sections were counterstained with To-pro-3 (Molecular Probes, Eugene, OR, USA) and mounted in Fluoromount (Leica, Wetzlar, Germany). Nuclei were counterstained with DAPI (Thermo Fisher, Waltham, MA, USA, dilution 1:1000). Images were acquired under the Leica TCS SP2 (Leica, Wetzlar, Germany) confocal laser-scanning microscope using ×20 objective lenses, and images were acquired with Leica Application Suite XV3.7.6.25997 software (Leica microsystems CMS GmbH).

### 2.14. Western Blot

RPTEC and tARPCs cells were detached and centrifuged to obtain cellular pellets and then lysed with RIPA lysis buffer containing a cocktail of inhibitors of phosphatases and proteases. After protein quantification by Bradford assay, proteins (25 µg) were separated in 4–15% polyacrylamide gel (Bio-Rad) and then transferred onto a polyvinylidene difluoride (PVDF) membrane (Trans-BlotTurbo Midi PVDF, 0.2 mM; Bio-Rad) by means of the Trans Blot Turbo (Bio-Rad) transfer system. The membranes were incubated overnight with the following primary antibodies: CYP1B1 (Rabbit, ab137562, ThermoFisher); β-actin (Mouse, #22181220, Sigma-Aldrich, St. Louis, MO, USA). Subsequently, the following secondary antibodies were used: goat anti-rabbit IgG-HRP (#170-6515, Bio-Rad, Hercules, CA, USA); goat anti-mouse IgG-HRP (#170-6516, Bio-Rad). The electrochemiluminescence (ECL) system was used to detect the binding of the antibodies according to the manufacturer’s instructions. The chemiluminescence signal was acquired using Chemidoc and quantified using the Image J software.

### 2.15. Statistical Analysis

Statistical analyses were performed using Prism version 7.0 (GraphPad Software Inc). We used Student’s t-test to evaluate statistical significance between 2 groups, whereas ANOVA was applied to determine statistically significancy differences between more than 2 groups. Data are expressed as mean ± SD, and results were considered as significantly different using P less than 0.05, as indicated in figure legends. For correlation measurements, statistical analysis was performed using the corr.test function in Psych Library with R (ver. 4.2.2) by applying spearman correlation test, data are indicated as Spearman’s rank correlation coefficient rs: −1 < ρ < 1 and *p* value < 0.05. ROC curves were generated using the Cancer Genome Atlas (TCGA) [21] and Genotype-Tissue Expression (GTEx) databases [22], reporting RNA sequencing expression data from tumoral tissue biopsy samples of 9736 tumors and 8587 normal samples from the TCGA and the GTEx projects, respectively. The method used was gene expression profiling interactive analysis using the standard processing pipeline [23]. Subdivision between patients with high and low CD133/CYP1B1 expression was performed based on the cutoff of 75% (high) and 25% (low). The Mantel–Cox test was used as a log-rank test for the hypothesis. The Cox PH Model was used for the hazard ratio, and the 95% confidence interval information has been included in the survival plot as dotted lines.

The method for differential analysis of RNA sequencing expression CYP1B1 data from tumoral tissue biopsy samples and normal samples of the TCGA and the GTEx projects is one-way ANOVA, using disease state (Tumor or Normal) as variable for calculating differential expression. The expression data were first log2(TPM+1) transformed for differential analysis and the log2FC is defined as median(Tumor)—median(Normal). Genes with higher |log2FC| values and lower q values than pre-set thresholds are considered differentially expressed genes. 

## 3. Results

### 3.1. tARPC Protect RPTEC from Cisplatin-Induced Apoptosis and Necrosis

**Figure 1:**
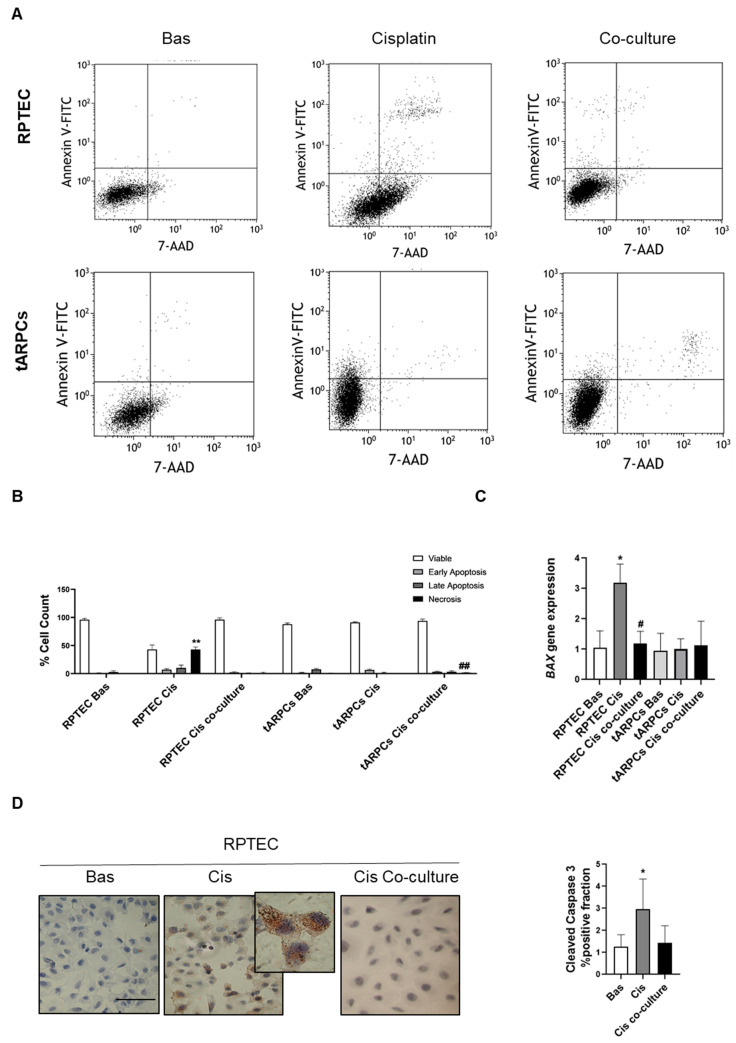
(**A**) Representative dot charts of annexin-V/7-AAD double-staining flow cytometry. RPTEC were treated with cisplatin at 2.5 mmol/L for 6h (condition RPTEC Cis) and then co-cultured for 96 h with tARPCs (RPTEC co-culture) seeded in a 0.4 mm polycarbonate insert after medium refresh (serum-free RPTEC medium). In another setting, tARPCs alone were stimulated by cisplatin for 6 h without RPTEC (tARPCs Cis). Flow cytometry assay analysis was performed at the end of 96 h. The lower-left quadrant (annexinV−/7-AAD−) contains viable cells, the upper-left quadrant (annexinV+/7-AAD−) represents early apoptotic cells, the upper-right quadrant shows late apoptotic cells (annexinV+/7-AAD+), and the lower-right quadrant (annexinV−/7-AAD+) represents necrotic cells. (**B**) The percentage of apoptotic cells in all conditions was statistically compared, and significant differences are denoted by ** *p* < 0.001 versus RPTEC basal condition and ## *p* < 0.001 versus RPTEC Cis. FITC, fluorescein isothiocyanate; 7-aminoactinomycin D; Cis, Cisplatin. Experiments are performed in triplicate, and data are expressed as the mean ± standard deviation of the mean (SD). (**C**) The pro-apoptotic gene BAX was significantly increased in RPTEC after Cis treatment and decreased following tARPCs co-culture (qPCR, mean ± SD, *n* = 5, * *p* < 0.05 versus RPTEC Basal, # *p* < 0.05 versus RPTEC Cis). (**D**) RPTECs apoptosis was assessed by positivity to caspase-3 4 days after 6 h of cisplatin treatment w/o tARPCs co-culture. (IHC, mean ± SD, *n* = 3, * *p* < 0.05), scale bar 50 µm.

Our group of research has previously shown that tARPC are cisplatin-resistant [12,19]. Furthermore, tARPC are able to preserve RPTEC proliferation rate during cisplatin-induced nephrotoxicity [12]. In order to better characterize the specific phase of cell death inhibition, early/late apoptosis and necrosis have been monitored using the annexin V-7AAD test. As shown in Figure 1A, RPTEC exposed for 6 h to cisplatin underwent high necrosis (43.05% ± 4.33% compared to basal 0.53% ± 0.27%, *p* < 0.05) with increased early apoptosis (7.04% ± 4.33%) and late apoptosis (10.33% ± 4.72%) compared to untreated cells. Conversely, the RPTEC stimulated for 6 h with cisplatin and then co-cultured with tARPC for 96 h displayed significantly fewer necrotic events (1.02% ± 1.33% vs. 43.05% ± 4.33% in the condition without the co-culture). In accordance, the apoptotic cells also decreased, particularly for the late apoptotic events (1.15% ± 0.12% co-culture vs. 10.35% ± 4.72% cisplatin). To further confirm apoptosis occurrence and modulation by tARPC, we assessed expression levels of pro-apoptotic gene *BAX* using real-time PCR. We found an increased gene expression of *BAX* 6 h after cisplatin exposition that significantly decreased in co-culture with tARPC. Instead, *BAX* expression did not increase in ARPCs stimulated with cisplatin, confirming the resistance of these cells to this chemotherapeutic agent. These findings were also confirmed by immunostaining for cleaved caspase-3 by using a specific antibody which detects endogenous levels of the large fragment (17/19 kDa) of activated enzyme. We found that the apoptosis rate significantly increases in cisplatin-damaged RPTEC compared to the basal condition, while the co-culture condition showed a lower apoptotic rate, suggesting a protective mechanism exerted by tARPC against cisplatin-induced damage.

### 3.2. Transcriptomic Analysis Revealed That tARPC Induce Overexpression of CYP1B1, a Cisplatin-Inactivating Enzyme

**Figure 2:**
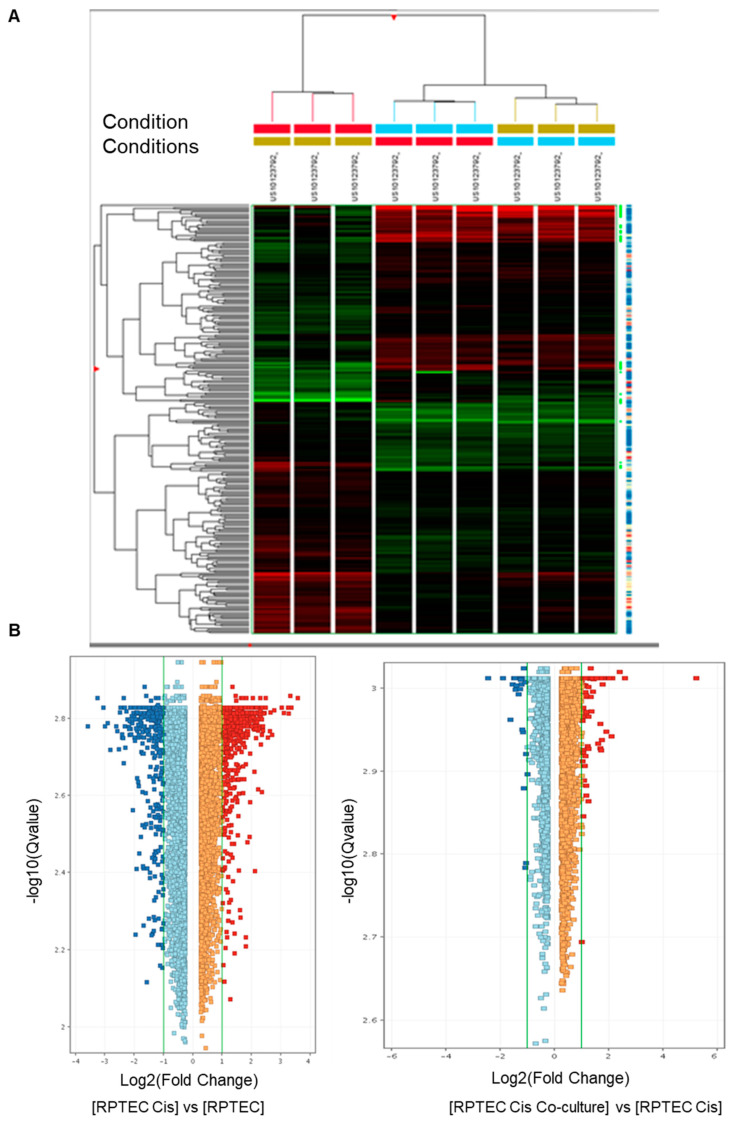
(**A**) Two-dimensional hierarchical clustering analysis with the visual alignment of metadata. The colors in the heatmap indicate the z-score. The red color indicates negative z-score, the black color indicates zero z-score, whereas the green color indicates positive z-score. Higher intensity of the color in the scale indicates a higher magnitude of the z-score. Clustering analysis allowed to group all samples and conditions of the dataset—(i) basal, (ii) cisplatin 6 h, (iii) cisplatin 6 h followed by tARPC co-culture—into subsets based on the similarity of their abundance profiles. (**B**) Scatter plots showing the modulated genes between (i) basal versus (ii) cisplatin 6 h (**left**) and (ii) cisplatin 6 h versus (iii) cisplatin 6 h followed by tARPC co-culture (**right**).

To gain insights into the molecular mechanism involved in tARPC’s protective and reparative effect against cisplatin nephrotoxicity, we performed a whole-genome transcriptomic analysis in RPTEC comparing three conditions: (i) basal condition (RPTEC Bas), (ii) RPTEC damaged by cisplatin for 6 h (RPTEC Cis), and (iii) RPTEC damaged by cisplatin for 6 h followed by co-culture with tARPC (RPTEC co-culture). The aim of this approach was to dissect genes specifically induced by tARPC following cisplatin toxicity. Firstly, via hierarchical clustering analysis, we found that all three conditions could be distinguished by their gene expression profiles independently. A main cluster separated cisplatin-damaged and cisplatin-damaged co-cultured RPTEC from the RPTEC in basal condition. However, a secondary cluster distinguished cisplatin-damaged RPTEC from damaged RPTEC in co-culture with tARPC (Figure 2A). Cisplatin treatment induced the modulation of 934 genes with a fold change of 2 and a false discovery rate (FDR) < 0.05 in RPTEC compared to basal condition. However, when the same treatment was followed by the tARPC co-culture, only 143 genes were found modulated in RPTEC (Figure 2B).

**Figure 3:**
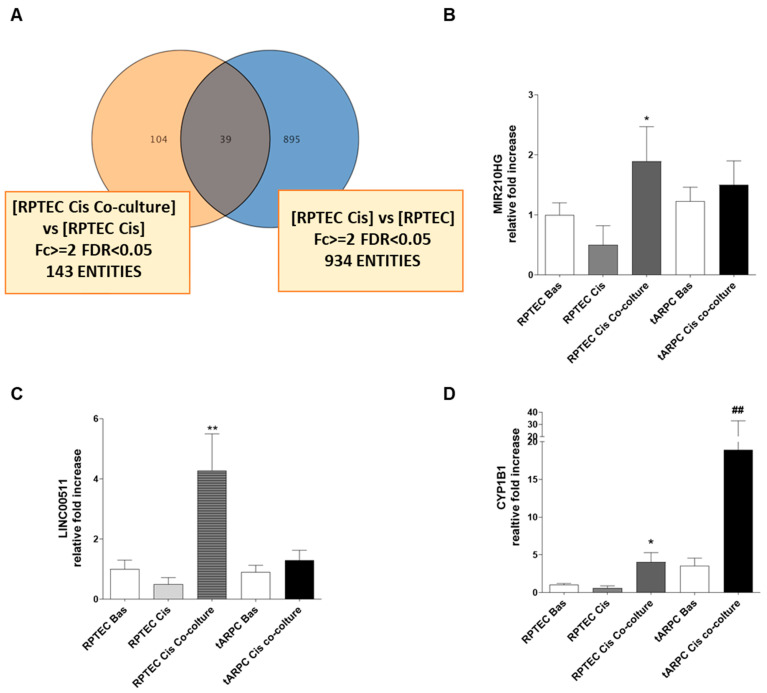
(**A**) Venn diagram showing the 39 genes specifically induced in RPTECs by tARPCs in co-culture following the cisplatin damage. (**B**–**D**) Real-time PCR validation of differentially expressed targets: (**B**) MIR210HG, (**C**) LINC00511, (**D**) CYP1B1. All three genes were significantly upregulated in RPTEC in co-culture conditions compared to cisplatin and basal conditions. (qPCR, mean ± SD, *n* = 5, * *p* < 0.05 versus RPTEC basal, ** *p* < 0.001 versus RPTEC basal, ## *p* < 0.001 versus tARPC basal).

Among these 143 genes, by overlapping differently modulated genes in all conditions, we identified 104 genes specifically induced in RPTEC by tARPC in co-culture following the cisplatin damage and only 39 genes in common with the 934 genes modulated in the comparison between RPTEC Cis and RPTEC (Figure 3A). Among the most upregulated genes induced by ARPCs, we found the CYP1B1 gene (FDR < 0.05; fold change = 4.34) the MIR210HG and LINC00511 (Table 2). Interestingly, CYP1B1 is directly linked to the cisplatin metabolism [24] and is involved in AKI [25]. Differential expression of these genes was validated via real-time PCR as shown in Figure 3B–D. In particular, we found that in co-culture, CYP1B1 expression in RPTEC shows a four-fold increase relative to basal, whereas it was almost undetectable in cisplatin-damaged RPTEC. Surprisingly, when we assessed CYP1B1 gene expression in tARPC co-cultured with cisplatin-damaged RPTEC, we detected a nearly 16-fold increase compared to RPTEC in co-culture and to untreated tARPC. (Figure 3D). This data suggests that the increased CYP1B1 gene expression was both induced by tARPC in cisplatin-damaged RPTEC and at the same time was strongly increased in tARPC themselves following sensing cisplatin-damaged RPTEC. MIR210HG and LINC00511 were also upregulated when damaged RPTEC were co-cultured with ARPC (Figure 3B,C). However, the highest gene expression upregulation in co-cultured tARPC was observed solely for CYP1B1. Therefore, we decided to deepen the mechanism related to this gene.

### 3.3. tARPC Induce Cisplatin Resistance in RPTEC by Releasing EVs Containing CYP1B1

**Figure 4:**
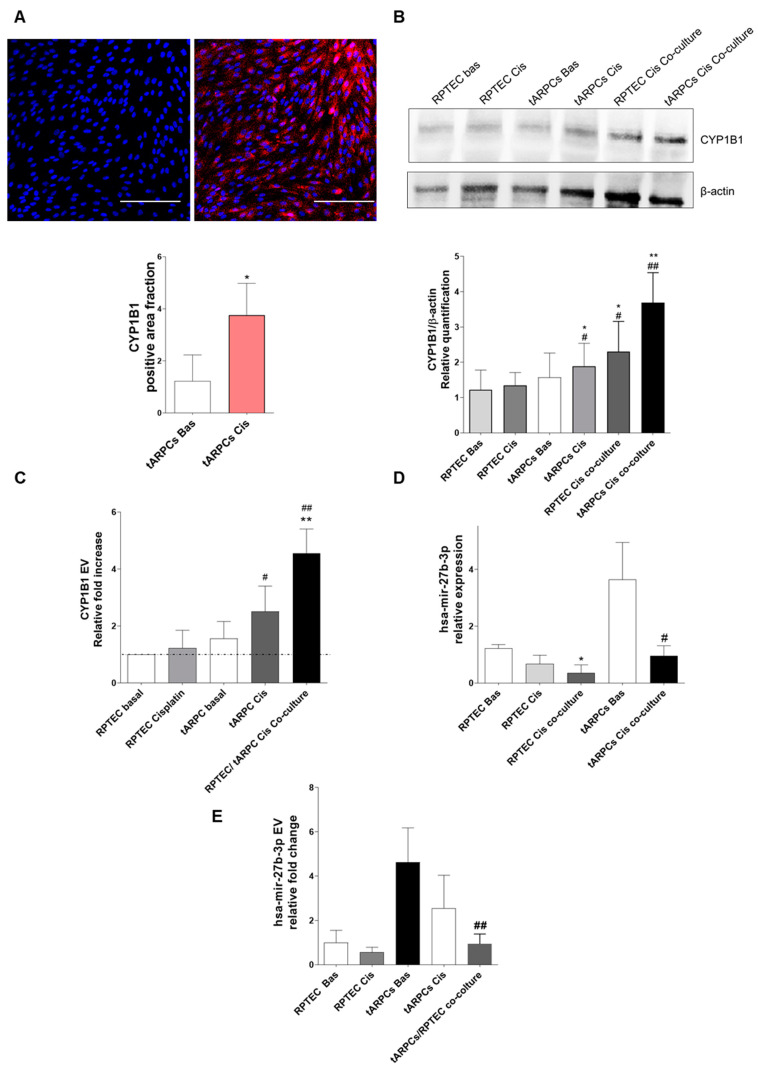
(**A**) Validation of the expression of CYP1B1 with immunofluorescence assay on cisplatin-treated tARPCs. CYP1B1 is widely expressed (red) after 6 h stimulation with cisplatin. Scale bar: 100 µm. (**B**) western blot analysis for CYP1B1 with relative quantification. CYP1B1 is significantly higher in the co-culture conditions (RPTEC Cis co-culture compared to RPTEC Bas and tARPCs Cis co-culture compared to tARPCs Bas); * *p* < 0.05 and ** *p* < 0.001 versus RPTEC basal, # *p* < 0.05 and ## *p* < 0.001 versus tARPCs basal. (**C**) CYP1B1 expression assessed in EV from medium culture of RPTEC, tARPCs and co-culture conditions. ** *p* < 0.001 versus RPTEC basal, # *p* < 0.05 and ## *p* < 0.001 versus tARPCs basal. (**D**) Real-time PCR for miR-27b-3p. In basal conditions, tARPC express significantly higher levels of miR-27b-3p compared to RPTEC basal, following co-culture with cisplatin damaged RPTEC level were significantly decreased; * *p* < 0.05, # *p* < 0.05 versus tARPCs basal. (**E**) miR-27b-3p levels are strongly decreased in co-culture condition compared to basal RPTECs and basal tARPCs (qPCR, mean ± SD, *n* = 5 *t*-test, ## *p* < 0.001). EV: extracellular vesicles.

Subsequently, we confirmed the expression of CYP1B1 protein via immunofluorescence and western blot. As expected, cisplatin-treated tARPC showed an increased expression of CYP1B1 compared to basal (Figure 4A, *p* < 0.05). Using western blot, we quantified the CYP1B1 protein level and found a more significant increase in tARPC co-cultured with RPTEC after cisplatin–induced damage. RPTEC were also able to increase CYP1B1 expression but solely in the presence of tARPC (Figure 4B), whereas—in accordance with gene expression findings—no significant increase was observed in RPTEC Cis (not shown). In co-culture experiments, tARPC and RPTEC were physically separated through the membrane of a Transwell. Therefore, to investigate the eventual role of extracellular vesicles (EV) in the cellular cross-talk, we performed EV isolation from culture and co-culture supernatants. Subsequently, we isolated and purified the total RNA from EVs, then performed real-time PCR for CYP1B1. We observed that CYP1B1 is highly expressed in the EV extracted from the supernatants of RPTEC/tARPC co-culture and is less so in tARPC exposed to cisplatin (Figure 4C) Collectively, these data indicate that CYP1B1 mRNA is strongly released by tARPC in response to cisplatin-damaged RPTEC through extracellular vesicles. In order to deepen the role of tARPC in modulating cisplatin-damaged RPTEC gene expression, we studied the correlation between CYP1B1 and its validated regulatory miRNAs, namely miR-27b-3p. Interestingly, miR-27b-3p is highly expressed in basal tARPC, while it is down-regulated in co-culture with cisplatin-damaged RPTEC (Figure 4D). In addition, we evaluated miR-27b-3p levels in EV from the medium of RPTEC and tARPC culture conditions, including the co-culture, and we observed a comparable trend of expression observed in RNA from cellular lysates. (Figure 4E). Overall, our data indicate that following sensing the cisplatin RPTEC damage, tARPC upregulated the CYP1B1 transcript through the miR-27b-3p modulation and then released CYP1B1 mRNA as cargo in their released EV. These events may contribute to cisplatin inactivation and the survival of renal epithelium during an episode of cisplatin nephrotoxicity.

### 3.4. CYP1B1 Is Expressed in Urinary Extracellular Vesicles from Cisplatin-Treated Patients with Nephrotoxicity and Showed Co-Localization with CD133^+^ tARPC

**Figure 5:**
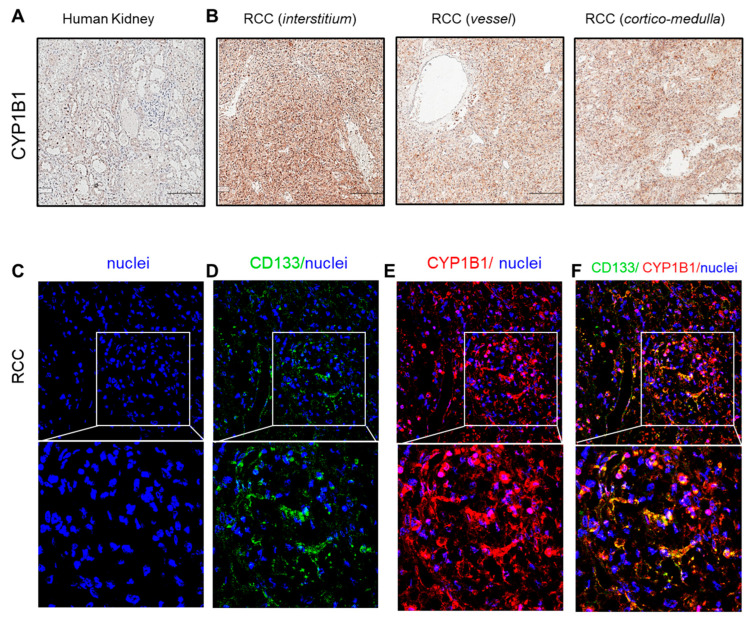
CYP1B1 staining on renal biopsies of renal cell carcinoma (RCC) compared to normal human kidney. (**A**,**B**) CYP1B1 immunochemistry; (**A**) human health kidney shows a weak cytoplasmic tubular staining, indicating a low expression of CYP1B1 in normal tissue; (**B**) renal cell carcinoma (RCC) shows loss of the typical renal tissue architecture and displays strong cytoplasmic staining, confirming the upregulation of CYP1B1 in cisplatin-treated patients at the level of the interstitium (**left**), vessel compartment (**middle**), and cortico-medulla region (**right**), Scale bar: 200 µm. (**C**–**F**) Immunofluorescence for CYP1B1/CD133 on renal cell carcinoma (RCC) biopsy. (**C**) Topro-3-labelled nuclei; (**D**) CD133 expression (green); (**E**) CYP1B1 expression (red); (**F**) CYP1B1 and CD133 expression (merge, yellow). Topro-3 was used to counterstain nuclei (blue), (magnification 20×). Enlargements of the boxed area of (**C**–**F**) are displayed in the lower row (magnification 63×).

To assess the expression of CYP1B1 ex vivo, we performed immunohistochemistry assays on biopsies of healthy human kidney tissue and cisplatin-treated renal cell carcinoma (RCC) (Figure 5A,B). As expected [26], CYP1B1 is highly expressed in renal carcinoma (Figure 5B), whereas healthy kidney tissue displays a low expression of this protein (Figure 5A). In order to detect the possible involvement of tARPC in CYP1B1 expression, we performed an immunofluorescence assay on RCC biopsies for CD133 and CYP1B1 (Figure 5D–F). We found that almost all CD133^+^ cells produced high quantities of CYP1B1 protein, as shown by a double positive CD133/CYP1B1 cell population. This is evident also at the tubular level, where the co-localization of the two markers clearly outlined tubular structures. This confirmed the wide expression of CYP1B1 in tumor tissues by tARPC as a response to the damage caused by cisplatin. 

Then, in order to understand whether CYP1B1 levels could correlate with clinical cisplatin nephrotoxicity, we evaluated the CYP1B1 expression from urinary EV collected from oncological patients with nephrotoxicity. The clinical characteristics of patients are listed in Table 2.

**Figure 6:**
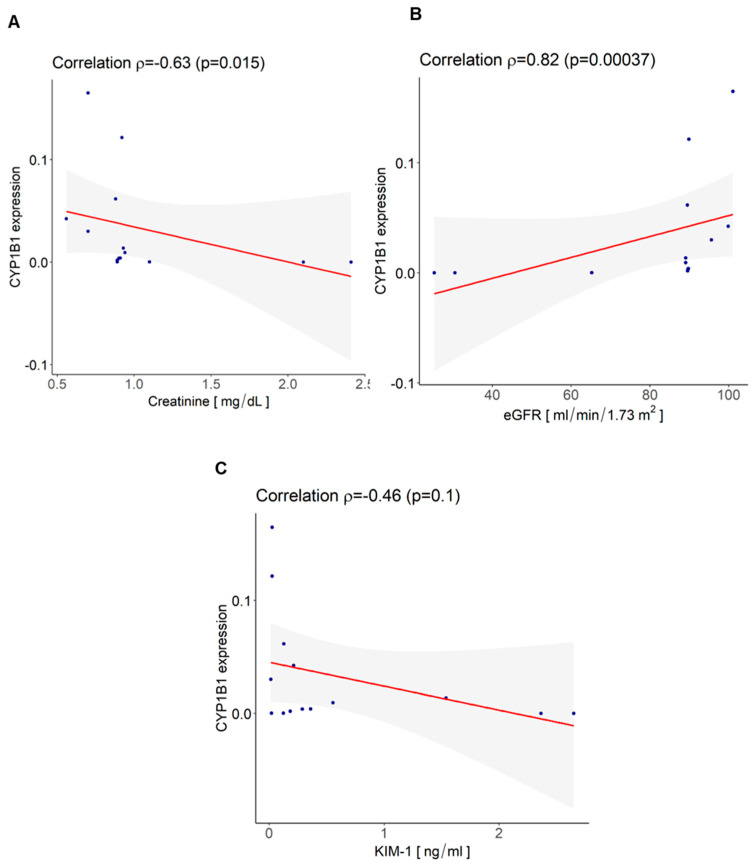
Correlation between urinary EV mRNA CYP1B1 levels and parameters of renal function. (**A**) Significant inverse correlation between urinary EV mRNA CYP1B1 level and creatinine (mg/dL) (ρ = −0.63 and *p* = 0.015); (**B**) significant positive correlation between urinary EV mRNA CYP1B1 level and eGFR (mL/min/1.73 m^2^) (ρ = 0.82.and *p* = 0.00037); (**C**) no significant correlation was observed with level of KIM-1 (ng/mL) even if an inverse trend was observed. Statistical analysis was performed using the corr.test function in Psych Library with R (ver. 4.2.2); Spearman correlation test, shaded areas represent 95% confidence interval.

Interestingly, we found a positive correlation between urinary EV mRNA CYP1B1 level and eGFR (mL/min/1.73 m^2^) (*ρ* = 0.82 and *p* = 0.00037) and a significant inverse correlation between urinary EV mRNA CYP1B1 level and creatinine (mg/dl) (*ρ* = −0.63 and *p* = 0.015) (Figure 6A,B), whereas no significant correlation was observed with the level of KIM-1 even if an inverse correlation trend was found (Figure 6C). These data confirmed the CYP1B1-protective effect of renal function.

**Figure 7:**
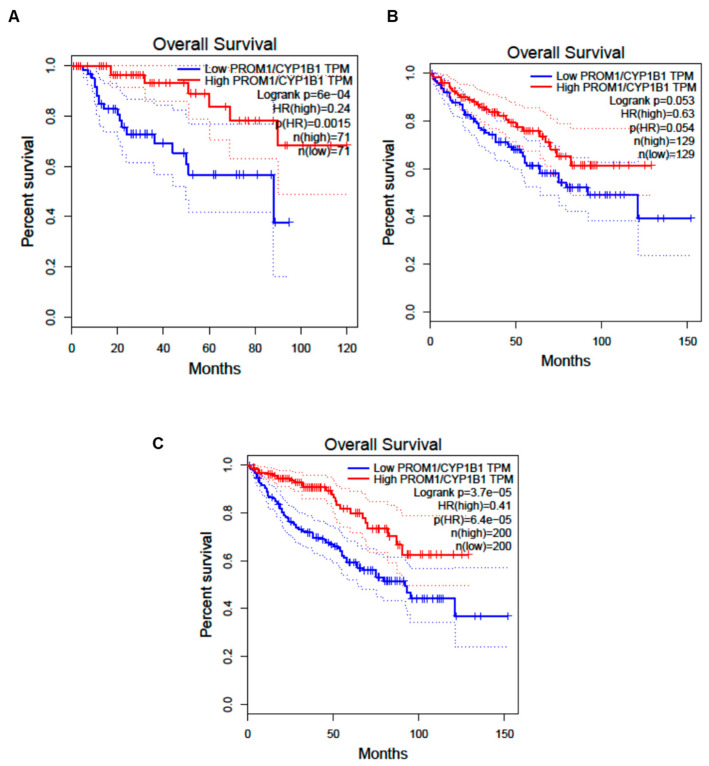
Overall survival analysis of patients with renal clear cell and renal papillary cell carcinoma based on the CD133/CYP1B1 expression. We performed this ROC curve analysis using the Cancer Genome Atlas (TCGA) [21] and Genotype-Tissue Expression (GTEx) databases [22], which report RNA sequencing expression data of 9736 tumors from tumoral tissue biopsy samples, including renal clear cell and renal papillary cell carcinoma. (**A**) ROC curve showing that renal clear cell carcinoma patients with higher renal CD133/CYP1B1 expression had a better outcome compared to those with lower CD133/CYP1B1expression. (**B**) ROC curve showing that renal papillary cell carcinoma patients with higher renal CD133/CYP1B1 expression had a better outcome compared to those with lower CD133/CYP1B1 expression. (**C**) ROC curve cumulative for renal clear cell carcinoma patients and renal papillary cell carcinoma showed that patients with higher renal CD133/CYP1B1 expression had a better outcome compared to those with lower CD133/CYP1B1expression. Subdivision between patients with high and low CD133/CYP1B1 expression was performed based on the cutoff of 75% (high) and 25% (low). Dotted lines represent the 95% confidence intervals. TPM, transcripts per million; HR, hazard ratio; *p*(HR), *p* value of Hazard ratio; *n*, number of analyzed samples; Logrank, log-rank test. ROC curves were generatd by Gene Expression Profiling Interactive Analysis [23].

Finally, we interrogated the TCGA and the GTEx database analyzing the CYP1B1 RNA sequencing expression data of 9736 tumors and 8587 normal samples from projects using a standard processing pipeline [23]. We found that the expression of CYP1B1 was quite heterogeneous among the several types of tumors and that it is often lower in normal tissues compared to tumoral tissues, as in the case of renal cancers (Supplemental Figures S1 and S2). Moreover, we performed an overall survival analysis of patients with two different renal cancers (renal clear cell and renal papillary cell carcinoma) based on the renal Prominin 1 (CD133) gene expression normalized on the total CYP1B1 gene expression. Interestingly, we found that carcinoma patients with higher renal CD133/CYP1B1 expression had better outcomes compared to those with lower CD133/CYP1B1 expression (*p* = 0.05 and *p* = 6 × 10^−4^, respectively; Figure 7A,B). These results were also confirmed by the ROC curve cumulative for the two types of renal cancer (*p* = 3.7 × 10^−5^, Figure 7C), suggesting that higher ARPC presence is correlated with improved overall survival.

## 4. Discussion

In this study, we investigated the molecular mechanisms underlying the renoprotective effect of tARPC on cisplatin-damaged RPTEC. We found that cisplatin affects RPTEC viability by inducing early and late apoptosis, whereas tARPC were able to completely revert this process. Additionally, tARPC were capable of modifying RPTEC transcriptome by inducing overexpression of CYP1B1, an enzyme that inactivates cisplatin, conferring resistance during chemotherapy; furthermore, we found that this event was regulated in a paracrine manner by tARPC-released EV. EV-derived tARPC may represent a promising strategy for alleviating nephrotoxicity in patients before undergoing cisplatin chemotherapy. Cisplatin is an antineoplastic drug used in the treatment of many solid-organ cancers. Despite extensive efforts to find alternative treatments less toxic and equally effective over the years, cisplatin remains the most commonly prescribed anticancer drug in treatment regimens for head and neck cancers, non-small cell lung cancer, ovarian and cervical cancer, bladder cancer, and others [1,6,10,27]. Cisplatin-induced nephrotoxicity is the main adverse effect of cisplatin-based chemotherapy and highly limits its clinical use [3,5,28]. Mechanisms of cisplatin-induced nephrotoxicity are complex and involve multiple pathways and molecules. However, the final common pathway is represented by tubular apoptosis induction. Remarkably, very recently, the regenerative activity of adult renal stem/progenitor cells (ARPCs) has been reported [29,30,31].

tARPC promote kidney regeneration in two ways: by directly differentiating [30,32] and by secreting reparative molecules. ARPCs have been shown to be able to regenerate lengthy segments of renal tubules and missing podocytes in cortical nephrons after AKI [18,33]. Firstly, we observed that under co-culture conditions, tARPC protect RPTEC from cisplatin-induced apoptosis. These findings are consistent with our previous studies concerning tARPC protective role on tubular cell proliferation [11,12].

**Figure 8:**
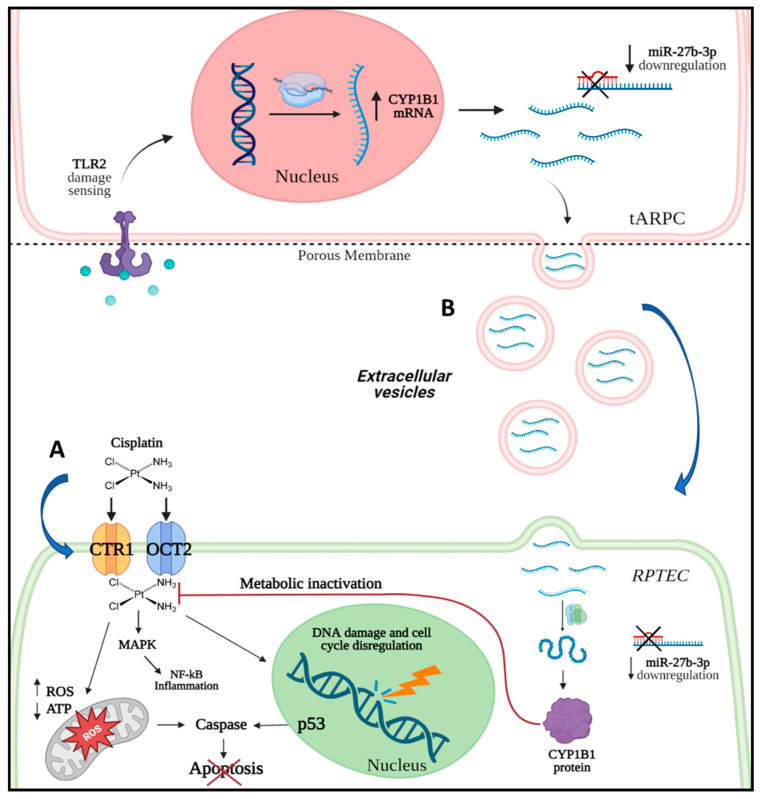
The release of CYP1B1 extracellular vesicles (EV) by tARPC induces cisplatin resistance in RPTEC. (**A**) Cisplatin enters RPTEC (green cell) through transmembrane transporters CTR1 and OCT1 and exerts its cytotoxic effect by activating NF-kB pro-inflammatory pathways, inducing mitochondrial damage, DNA damage, and cell cycle dysregulation; increasing ROS production; and ultimately leading to apoptosis. Through TLR2, tARPC (pink cells) are able to detect the injured RPTEC and to induce the up-regulation of the CYP1B1 gene and down-regulation of its validated regulatory miRNA, miR-27b-3p. The blue dots represent the DAMPs—“damage-associated molecular pattern molecules”, also termed “alarmins”—associated with cisplatin-induced renal damage. (**B**) The mRNA of CYP1B1 is then shuttled by tARPC-released EV and delivered into recipient cells, leading to up-regulation of CYP1B1 expression. As a consequence of this process, cisplatin is deactivated, and RPTEC becomes resistant to cisplatin due to a reduction in apoptosis.

Using a whole-genome approach, we were able to identify all differentially regulated genes by tARPC during cisplatin nephrotoxicity, specifically the CYP1B1 gene, which has an important role in cisplatin detoxification. Our data indicate that following sensing the cisplatin RPTEC damage, tARPC upregulated the CYP1B1 transcript (16-fold increase in tARPC co-cultured with cisplatin-damaged RPTEC) through the miR-27b-3p modulation and then release CYP1B1 mRNA as cargo in their released EV. As previously shown, ARPC can detect cisplatin damage via toll-like receptor 2 (TLR-2); the blocking of this receptor prevented the protective role of ARPC against cisplatin [12] (Figure 8).

CYP1B1 is an extrahepatic member of the cyto-chrome P450 that catalyzes the metabolism of several xenobiotics. It has been shown to be capable of metabolizing anticancer drugs such as docetaxel, cisplatin, tamoxifen, and nucleoside analogs [24] and is involved in AKI since the complement system induced a significant CYP1B1 level increase, modulating its gene DNA methylation [25,34,35]. 

At renal level, the CYP1B1 overexpression induced in RPTEC by tARPC can be interpreted as an attempt to protect RPTEC from cisplatin-induced nephrotoxicity and may open an important strategy of prevention of AKI before starting chemotherapy. 

Recently, the possibility of administering EV has gained much interest in kidney-related diseases. In addition to its ability to maintain or mimic stem cells’ effects, the advantage of these cell-free agents bypassing most of the safety concerns related to stem cell-based therapy (tumorigenic potential or rejection) has paved the use of EV in clinical trials [36]. Our finding that tARPC can release CYP1B1 through EV can offer the possibility of administering tARPC EV as renoprotective strategy during chemotherapy. 

In this study, we investigated the presence of a particular miRNA, miR-27b-3p, which is responsible for the regulation of CYP1B1 [19,37,38,39,40] and is involved in renal diseases [41]. In breast cancerous tissues miR-27b-3p level is decreased compared to non-cancerous tissues, leading to the high expression of CYP1B1 [42,43]. We found that miR-27b-3p is highly expressed in basal tARPC, while it results in down-regulation in co-culture with cisplatin-damaged RPTEC. A similar trend of expression was observed in EV from medium of various culture conditions, coherently with the inverse expression levels of CYP1B1 in EV. 

In addition, we showed that the lncRNAs MIR210HG and LINC00511 were up-regulated in tARPC/RPTEC co-cultures after cisplatin exposition compared to RPTEC. Even if the exact role of these lncRNAs in the context of nephrotoxicity is not well-understood, several studies provided evidence toward them providing a supporting function to CYP1B1. Indeed, MIR210HG has been found to potentiate the hypoxia-inducible factor 1α, which is activated by hypoxia [44]. In turn, hypoxia upregulates CYP1B1 [45]. However, LINC00511 has been found to support cisplatin resistance in non-small-cell lung cancer by elevating miR-625 level [46]. 

Most studies agree that CYP1B1 protein is commonly overexpressed in malignant as compared to normal tissue, conferring resistance to prolonged chemotherapy (i.e, as paclitaxel and docetaxel) [47,48,49,50,51]. However, CYP1B1’s precise role in drug resistance remains obscure. Regarding CYP1B1 significance at the tumor level, CYP1B1 was associated both with protective overall survival in skin cutaneous melanoma and sarcoma, whereas its expression correlates with tumor stage in bladder urothelial carcinoma and other tumors [26]. Here, we found a co-localization between the CYP1B1 and CD133 marker in RCC biopsies of cisplatin-treated patients, confirming the hypothesis of a potential renoprotective role of this enzyme during chemotherapy. The CD133/CYP1B1 renoprotective role has been also showed by our overall survival analysis on 200 renal cancer patients, highlighting a better outcome for patients expressing high levels of ARPC. 

Moreover, in our cohort of oncological patients treated with cisplatin or other analogues, we demonstrated a positive correlation between level urinary EV CYP1B1 and eGFR, indicating that level of this detoxifying enzyme—mainly released by tARPC—could predict occurrence of nephrotoxicity, thereby counteracting tubular necrosis. This kind of mechanism may be common to several platinum-based drugs. We did not observe significant differences between patients treated with cisplatin, oxaliplatin, or carboplatin, but further studies are needed to understand this matter as well as the precise source of urinary EV containing CYP1B1. Urinary EV offer potential biomarkers of AKI [52,53], renal rejection [20], and regeneration [54] even if further studies are required for their precise source characterization. 

In the near future, purification of urinary tARPC and their RNA cargo characterization in conjunction with serum creatinine and eGFR could offer a potential strategy for stratifying patients at higher risk of nephrotoxicity. 

We acknowledge that this study has limitations. Mainly, the number of patients with adenocarcinoma diagnoses (colon, lung, stomach, head and neck cancer, and breast cancer) enrolled was small. However, we were able to validate the findings of this study on ten different human and primary tARPC lines isolated from renal tissues with functional experiments and, furthermore, on a cohort of 200 cancer patients from external databases. Moreover, in many kinds of cancer, the increase in CYP1B1 expression can be due to the tumor itself, and we cannot be sure that in RCC its expression is due to cisplatin treatment. However, when we performed an analysis of CYP1B1 expression in tumor tissue and its normal counterpart deriving from the same subject, we found that CYP1B1 is more expressed in normal tissue than in tumoral tissue (Supplemental Figures S1 and S2). This in silico analysis, together with our experimental data showing that CYP1B1 expression increase following cisplatin stimulation, support the hypothesis that the increase in CYP1B1 is due to cisplatin treatment and not the tumor itself. 

In conclusion, we revealed a new mechanism by which the tARPC, which are resistant to cisplatin, can protect RPTEC from cisplatin-induced apoptosis via the upregulation of CYP1B1 gene transcript and its consequent release in circulating EV. Concomitantly to the increased CYP1B1 expression, we assessed the decreased EV level of the validated inhibitory miR-27b-3p. This mechanism can be activated through the binding of damage-associated molecular pattern molecules, associated with cisplatin-induced renal damage, to the toll-like receptor 2 expressed on tARPC [12].

Thus, ARPC-released CYP1B1 in EV represents a defense mechanism against cisplatin toxicity, acting as a detoxifier in damaged cells and determining resistance to the anti-neoplastic drug. This mechanism may justify the different susceptibilities of patients to cisplatin-induced AKI as nephrotoxicity that could be related to the abundance of renal progenitors, which is highly variable among different patients—differing by age, sex, and habits—and may open new therapeutic strategies or allow the use of tARPC released-CYP1B1 as potential predictor of nephrotoxicity risk in the future. 

## Figures and Tables

**Table 1:** Clinical features of oncological patients.

Variables	Values
Male	70% (*n* = 10)
Female	30% (*n* = 4)
Age (years)	66.7 ± 7.9
Reason for Chemotherapy	colon adenocarcinoma (*n* = 6)
	lung adenocarcinoma (*n* = 2)
	stomach adenocarcinoma (*n* = 2)
	head and neck cancer (*n* = 2)
	breast cancer (*n* = 2)
Creatinine (mg/dL) mean ± SD	1.07 ± 0.65
eGFR (mL/min/1.73 m^2^) mean ± SD	79.26 ± 24.16
	Oxaliplatin (*n* = 6)
Treatments	Cisplatin (*n* = 8)
	Carboplatin (*n* = 1)

**Table 2:** List of top up-regulated and down-regulated genes in RPTEC/tARPC Cis conditions compared to RPTEC Cis.

Up-Regulated Genes			
ProbeName	Fold Change	GeneSymbol	Description
A_23_P33759	37.55951	DHRS3	Homo sapiens dehydrogenase/reductase (SDR family) member 3 (DHRS3). mRNA [NM_004753]
A_23_P416965	6.0632515	FAM149A	Homo sapiens family with sequence similarity 149. member A (FAM149A). transcript variant 1. mRNA [NM_015398]
A_23_P349406	5.2752495	RIMKLA	Homo sapiens ribosomal modification protein rimK-like family member A (RIMKLA). mRNA [NM_173642]
A_33_P3290343	4.349999	CYP1B1	Homo sapiens cytochrome P450. family 1. subfamily B. polypeptide 1 (CYP1B1). mRNA [NM_000104]
A_22_P00005242	4.348923	lnc-DLX2-4	LNCipedia lincRNA (lnc-DLX2-4). lincRNA [lnc-DLX2-4:3]
A_23_P209625	4.2065525	CYP1B1	Homo sapiens cytochrome P450. family 1. subfamily B. polypeptide 1 (CYP1B1). mRNA [NM_000104]
A_23_P81158	4.171087	ADH1C	Homo sapiens alcohol dehydrogenase 1C (class I). gamma polypeptide (ADH1C). mRNA [NM_000669]
A_23_P35414	3.626841	PPP1R3C	Homo sapiens protein phosphatase 1. regulatory subunit 3C (PPP1R3C). mRNA [NM_005398]
A_22_P00016430	3.2619064	HIF1A	HIF1A antisense RNA 2 [Source:HGNC Symbol;Acc:HGNC:43015] [ENST00000554254]
A_23_P208937	3.2162015	TLE6	Homo sapiens transducin-like enhancer of split 6 (TLE6). transcript variant 2. mRNA [NM_024760]
A_23_P132027	3.0906937	SPAG4	Homo sapiens sperm associated antigen 4 (SPAG4). mRNA [NM_003116]
A_24_P362904	3.0134628	PFKFB4	Homo sapiens 6-phosphofructo-2-kinase/fructose-2.6-biphosphatase 4 (PFKFB4). mRNA [NM_004567]
A_33_P3247175	2.9417257	C4orf47	Homo sapiens chromosome 4 open reading frame 47 (C4orf47). mRNA [NM_001114357]
A_22_P00014909	2.8318717	TPBGL	Homo sapiens trophoblast glycoprotein-like (TPBGL). mRNA [NM_001195528]
A_22_P00002306	2.7089448	MIR210HG	Homo sapiens MIR210 host gene (non-protein coding) (MIR210HG). long non-coding RNA [NR_038262]
A_21_P0009360	2.4929426	LINC00673	Homo sapiens long intergenic non-protein coding RNA 673 (LINC00673). long non-coding RNA [NR_036488]
A_22_P00014796	2.3685126	LINC00673	Homo sapiens long intergenic non-protein coding RNA 673 (LINC00673). long non-coding RNA [NR_036488]
A_24_P37441	2.3357887	PDK1	Homo sapiens pyruvate dehydrogenase kinase. isozyme 1 (PDK1). transcript variant 2. mRNA [NM_002610]
A_23_P76071	2.2893877	B3GNT4	Homo sapiens UDP-GlcNAc:betaGal beta-1.3-N-acetylglucosaminyltransferase 4 (B3GNT4). mRNA [NM_030765]
A_23_P24077	2.2306385	C10orf54	Homo sapiens chromosome 10 open reading frame 54 (C10orf54). mRNA [NM_022153]
A_24_P237586	2.2281685	ANKRD37	Homo sapiens ankyrin repeat domain 37 (ANKRD37). mRNA [NM_181726]
A_21_P0011646	2.137652	LINC00511	Homo sapiens long intergenic non-protein coding RNA 511 (LINC00511). long non-coding RNA [NR_033876]
A_23_P347610	2.0365067	HAVCR1	Homo sapiens hepatitis A virus cellular receptor 1 (HAVCR1). transcript variant 1. mRNA [NM_012206]
A_33_P3420416	2.0136702	LGALS9	Homo sapiens lectin. galactoside-binding. soluble. 9 (LGALS9). transcript variant 2. mRNA [NM_002308]
A_23_P16058	2.0103858	ZNF296	Homo sapiens zinc finger protein 296 (ZNF296). mRNA [NM_145288]
**Down-Regulated Genes**			
**ProbeName**	**Fold Change**	**GeneSymbol**	**Description**
A_22_P00006779	−2.0106018	FXR1	Homo sapiens fragile X mental retardation. autosomal homolog 1 (FXR1). transcript variant 3. mRNA [NM_001013439]
A_24_P376129	−2.0714304	DFNB31	Homo sapiens deafness. autosomal recessive 31 [Source:HGNC Symbol;Acc:HGNC:16361] [ENST00000374057]
A_33_P3261054	−2.1762295	CCDC114	Homo sapiens coiled-coil domain containing 114 (CCDC114). mRNA [NM_144577]
A_32_P171061	−2.5465722	ASCL2	Homo sapiens achaete-scute family bHLH transcription factor 2 (ASCL2). mRNA [NM_005170]

## Data Availability

The Agilent microarray data are Minimum Information About a Microarray Experiment (MIAME)-compliant, and raw data have been deposited in the database of the European Bioinformatics Institute (EMBL-EBI) and are accessible through Experiment ArrayExpress accession number E-MTAB-11451.

## Supplementary Materials

The following supporting information can be downloaded at: https://www.mdpi.com/article/10.3390/cells12121655/s1. Figure S1: CYP1B1 expression in Cancer. Figure S2: Differential Analysis of CYP1B1 RNA sequencing expression data from tumoral tissue biopsy samples and normal samples of the TCGA and the GTEx projects. Table S1: Primer Sequences and lnRNAs assay.
