# Supplementary material for: Human Adult Renal Progenitor Cells Prevent Cisplatin-Nephrotoxicity by Inducing CYP1B1 Overexpression and miR-27b-3p Down-Regulation through Extracellular Vesicles
Source: Cells. 2023 Jun 17;12(12):1655. doi: 10.3390/cells12121655 (PMC10296841; doi:10.3390/cells12121655)
Supplement: Supplementary file 1 [file cells-12-01655-s001.zip › cells-2437073-supplementary.pdf]

Supplementary Table S1

Primer Sequences and lnRNAs assay

CYP1B1	for: 5′-CCTCTTCACCAGGTATCC-3′<br>rev: 5′- ATGAGGAATAGTGACAGGC
BAX	For: 5′- TTTGCTTCAGGGTTTCATCC-3′<br>Rev: 5′-CAGTTGAAGTTGCCGTCAGA-3′
MIR210HG	RT² lncRNA qPCR Assay for Human MIR210HG (LPH15919A)<br>Entrez gene ID: 100506211<br>Detected transcript: NR_038262 (2303 bp)<br>ENST00000500447<br>Amplicon size: 84 bp
LINC00511	RT² lncRNA qPCR Assay for Human LINC00511 (LPH06350A)<br>Entrez gene ID: 400619<br>Detected transcripts<br>NR_033876 (2265 bp)<br>ENST00000580861<br>ENST00000581549<br>Amplicon size: 78 bp

Supplementary Figure S1

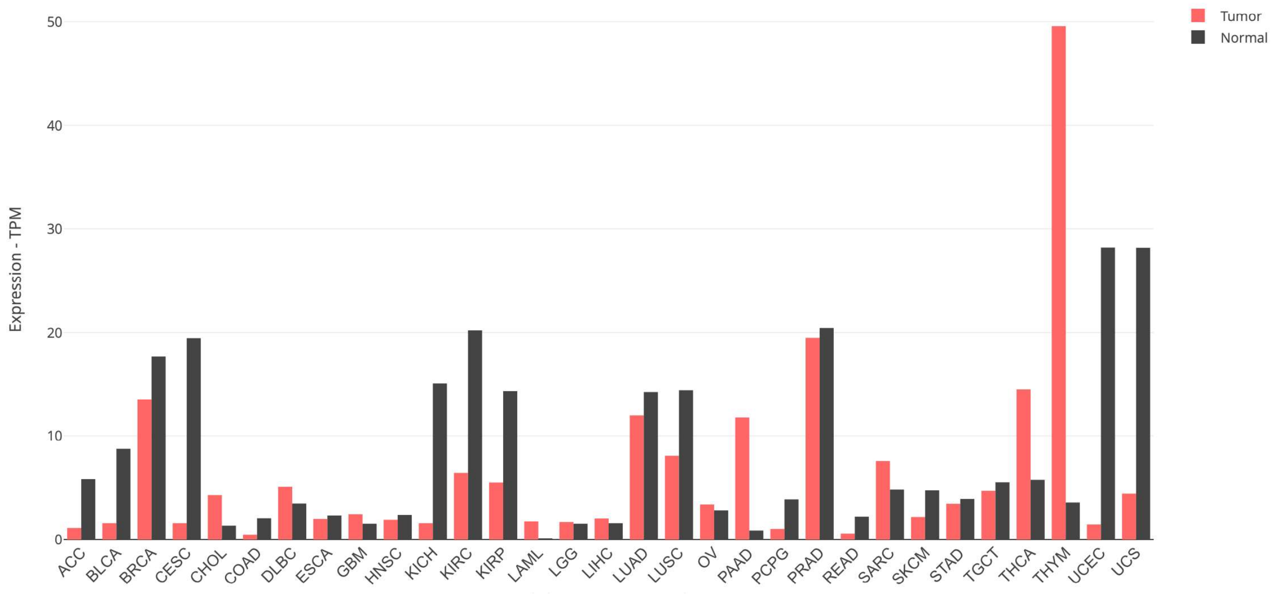

TCGA	Detail
ACC	Adrenocortical carcinoma
BLCA	Bladder Urothelial Carcinoma
BRCA	Breast invasive carcinoma
CESC	Cervical squamous cell carcinoma and endocervical adenocarcinoma
CHOL	Cholangio carcinoma
COAD	Colon adenocarcinoma
DLBC	Lymphoid Neoplasm Diffuse Large B-cell Lymphoma
ESCA	Esophageal carcinoma
GBM	Glioblastoma multiforme
HNSC	Head and Neck squamous cell carcinoma
KICH	Kidney Chromophobe
KIRC	Kidney renal clear cell carcinoma
KIRP	Kidney renal papillary cell carcinoma
LAML	Acute Myeloid Leukemia
LGG	Brain Lower Grade Glioma
LIHC	Liver hepatocellular carcinoma
LUAD	Lung adenocarcinoma
LUSC	Lung squamous cell carcinoma
MESO	Mesothelioma
OV	Ovarian serous cystadenocarcinoma
PAAD	Pancreatic adenocarcinoma
PCPG	Pheochromocytoma and Paraganglioma
PRAD	Prostate adenocarcinoma
READ	Rectum adenocarcinoma
SARC	Sarcoma
SKCM	Skin Cutaneous Melanoma
STAD	Stomach adenocarcinoma
TGCT	Testicular Germ Cell Tumors
THCA	Thyroid carcinoma
THYM	Thymoma
UCEC	Uterine Corpus Endometrial Carcinoma
UCS	Uterine Carcinosarcoma
UVM	Uveal Melanoma

Figure S1. CYP1B1 expression in Cancer. Bar plot showing the CYP1B1 expression in listed cancers and in relative normal tissue counterpart. TPM, transcripts per million; Bar plot was generated by generated by Gene Expression Profiling Interactive Analysis.

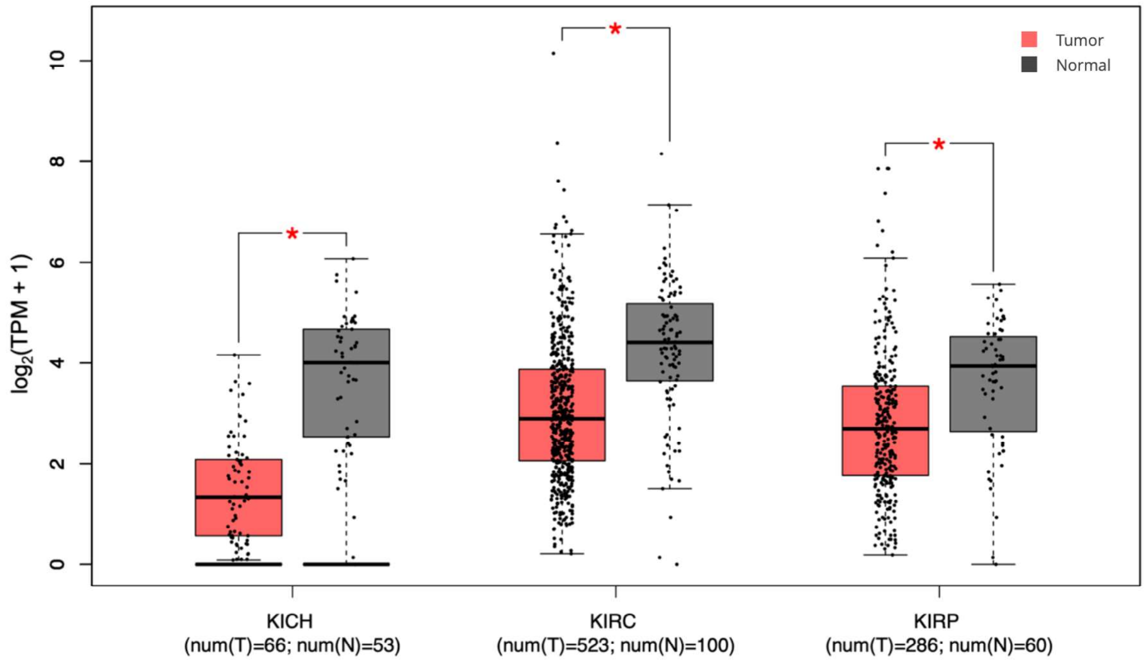

Supplemental Figure S2. Differential Analysis of CYP1B1 RNA sequencing expression data from tumoral tissue biopsy samples and normal samples of the TCGA and the GTEx projects. The CYP1B1 expression is lower in RCC tissue (Red box) than normal tissue (gray box). The method for differential analysis is one-way ANOVA, using disease state (Tumor or Normal) as variable for calculating differential expression. The expression data were first log2(TPM+1) transformed for differential analysis and the log2FC is defined as median(Tumor) - median(Normal). Genes with higher |log2FC| values and lower q values than pre-set thresholds are considered differentially expressed genes. TPM, transcripts per million; T, Tumor; N, Normal; KICH, kidney chomophobe carcinoma; KIRC, kidney renal clear cell carcinoma; KIRP, renal papillary cell carcinoma.
